# Stress-Affected Oxygen Reduction Reaction Rates on UNS S13800 Stainless Steel

**DOI:** 10.3389/fchem.2022.820379

**Published:** 2022-03-07

**Authors:** Carlos M. Hangarter, Rachel M. Anderson, Steven A. Policastro

**Affiliations:** Chemistry Division, U.S. Naval Research Laboratory, Washington, DC, United States

**Keywords:** galvanic corrosion, atmospheric corrosion, ORR, stress, strain, stainless-steel, mechano-electrochemistry

## Abstract

This work investigates the previously unexplored impact of tensile stress on oxygen reduction reaction (ORR) kinetics of a precipitation-hardened, stainless-steel fastener material, UNS S13800. ORR is known to drive localized and galvanic corrosion in aircraft assemblies and greater understanding of this reaction on structural alloys is important in forecasting component lifetime and service requirements. The mechano-electrochemical behavior of UNSS13800 was examined using amperometry to measure the reduction current response to tensile stress. Mechanical load cycles within the elastic regime demonstrated reversible electrochemical current shifts under chloride electrolyte droplets that exhibited a clear potential dependence. Strain ramping produced current peaks with a strain rate dependence, which was distinct from the chronoamperometric shifts during static tensile load conditions. Finally, mechanistic insight into the dynamic and static responses was obtained by deoxygenation, which demonstrated ORR contributions that were distinct from other reductive processes.

## Introduction

Mechanical stress and strain effects on reaction kinetics are well recognized for noble metal electrocatalyst materials (Amakawa et al., 2013; Du et al., 2015; Yan et al., 2016). For Pt catalysts, elastic strain has been deconvoluted from ligand effects using a NiTi shape memory alloy substrate. Compressive strain resulted in a 52% enhancement of the oxygen reduction reaction (ORR) kinetic rate while tensile strain led to a 35% decrease of the rate constant. These effects were attributed to changes in the overlap of adsorbate bonding and anti-bonding states with the Pt d-band. The mechanism was argued to originate from increased (compression) or decreased (tension) adsorbate bonding strength when the surface coverage of reaction intermediates was decreased or increased, respectively. Similar work has also been shown for Pd monolayers, in which lattice mismatch with various substrates is employed to induce compressive and tensile strain, to produce a linear correlation with hydrogen desorption potentials, in agreement with d-band theory (Kibler et al., 2005). Additionally, a large area of nanoparticle ORR catalyst research is based on lattice strains that arise from size and alloying effects, providing a clear and significant correlation between reaction kinetics and strain on clean metal surfaces (Strasser et al., 2010).

In contrast to noble metals, structural alloys are microstructurally heterogeneous and covered with relatively thick surface oxides, on the order of nanometers, that consequently yield a more complex electrocatalytic response to stress. Surface oxides are essentially dielectric, or semiconducting, thin films that span a range of conductivities. They are also subject to several distinct charge transfer mechanisms that influence their response to stress (Paola, 1989; Costa et al., 2014). Further, the passive films on structural alloys are not isomorphous. They range from amorphous to polycrystalline; typically falling within a nanocrystalline regime over alloy grains and have less order over intergranular regions (Maurice and Marcus, 2018). As a result, stress has multiple mechanistic routes to impact corrosion reaction kinetics (Chen et al., 2008; Thébault et al., 2011; Wang et al., 2014; Van den Steen et al., 2016; Hangarter C. and Policastro S. A., 2017; Hangarter C. M. and Policastro S. A., 2017; Liu et al., 2017; Palani et al., 2017; Alexander et al., 2018). Strain on pristine semiconductors induces density of states shifts that have been shown to impact charge carrier mobility (Sun et al., 2007). In addition, applied strains can drive defect formation and migration within the oxide, which the point defect model (Macdonald, 2011), has suggested can affect charge transfer rates at the metal-oxide interface. The complexity of the response of the oxide to strain suggests that there are multiple mechanisms occurring within the oxide. These can be observed, not only beyond the transition between elastic and plastic deformation of the oxide, but even within the expected elastic deformation region of the stress-strain curve for the oxide.

Vignal et al. (2001) used electrochemical impedance spectroscopy (EIS) studies to demonstrate elastic stress induced increased passive film conductivity for UNS S31600. These differences were argued to arise from film micro-plasticity above 70% of the yield strength. A separate report used a redox couple, Ru(NH_3_)_6_^3+/2+^, with a scanning electrochemical microscopy (SECM) feedback loop to quantify stress-affected charge transfer. That study examined UNS S31600 and found the heterogeneous kinetic constant decreased with applied tensile stress while the transfer coefficient remained unchanged (Sun et al., 2008). A similar SECM study on UNS S30400L yielded contradictory results with a different redox mediator, Fe(CN)_6_^3−/4−^. This discrepancy was attributed to adsorption differences in the supporting electrolyte (KNO_3_ vs. KSO_4_) as well as chemical and electronic structure differences of the passive film. Qualitative agreement with the latter was observed for acid treated UNS S30200 stainless steel springs under tensile stress, but the magnitude was greater, essentially doubling the kinetic constant. Taken as a whole, these reports indicate that the kinetic responses to elastic tensile stress for austenitic stainless steels is dependent on the material, pretreatment, and electrolyte due to passive film differences. In addition, there could also be opposing stress mechanisms at play in the elastic vs. plastic regimes (Sun et al., 2007; Sun et al., 2008; Svedruzic and Gregg, 2014; Maurice and Marcus, 2018).

The impact of stress and strain on the ORR kinetics is important from a galvanic corrosion perspective because aluminum alloys are commonly used for aircraft applications due to their high strength-to-weight ratio. However, because of the difficulty posed by the non-weldability of high-strength aluminum alloys, aircraft components are typically joined with fasteners (Boyer et al., 1984). These fasteners are usually made from stainless steel or titanium alloys that are more noble than the aluminum alloys used in airframe construction. Once electrical and ionic conductivity between the dissimilar metals is in place, galvanic corrosion can occur(Matzdorf et al., 2013; Feng et al., 2014; Feng and Frankel, 2014; Hangarter C. and Policastro S. A., 2017). This galvanic process consists of the ORR on the more noble material driving corrosion of the aluminum alloy, often at an accelerated rate. Prior work has shown that the galvanic corrosion in atmospheric environments can be more complex than what occurs under immersion conditions. Atmospheric environments are characterized by discontinuous electrolytes or droplets with unique physical and chemical constraints. These distinctions include an electrolyte that varies with temperature and humidity, with chloride concentrations from deliquescence to below seawater concentrations and oxygen diffusion lengths defined by electrolyte geometry (Schindelholz and Kelly, 2012; Liu et al., 2017; Marshall et al., 2019). The complexity of a galvanic atmospheric corrosion system is such that standard accelerated tests (e.g., ASTM B117) will often fail to produce the correct corrosion mechanisms and exhibit poor correlations with corrosion rates obtained from atmospheric exposures. Addressing the challenge of atmospheric corrosion requires targeted modeling and experimental approaches that can duplicate corrosion rates and reactions observed in relevant environments.

From an atmospheric perspective, the impact of chloride concentration on native oxide film charge transfer has yet to be examined. Recent work in dilute chloride electrolytes has demonstrated that chloride ions decreased passivity due to hindered enrichment of Cr^3+^, Mo^4+^ and Mo^6+^ content, while increasing hydroxylation in the passive film outer layer (Wang et al., 2020). This suggests the range of chloride concentrations experienced during galvanic atmospheric corrosion likely has an impact on passive film structure and charge transport. Importantly, stress-affected charge transfer studies discussed above have been limited to austenitic stainless steels. No work to date has specifically examined stress-affected kinetics on precipitation hardened martensitic alloys, which are typically used for high strength application in the aerospace industry. Moreover, while studies with redox mediators provide insight into charge transfer behavior, their redox potential is typically more positive than that required for ORR.

This work examines the effect of stress on cathodic current rates, to include ORR, for precipitation hardened stainless steel UNS S13800 PH. Chronoamperometry was used to monitor the effect of stress profiles on ORR current in real time. A droplet electrolyte cell in an environmental chamber was utilized to attain atmospheric corrosion conditions in high chloride concentrations. Two different experimental cell configurations were used for complementary oxygenated and deoxygenated experiments that enabled further analysis of the amperometric results.

## Materials and Methods

### Materials

The as-received UNS S13800 was precipitation hardened at 950°C with nominal composition indicated in Table 1. Sodium chloride (>99%), acetone (>99%) and isopropyl alcohol (>99%) (Fisher Scientific; Pittsburgh, PA, United States) were used without further purification.

**TABLE 1:** Nominal composition of UNS S13800.

Cr	Ni	Mo	Al	Mn	Si	C	N	P	S	Fe
12.75	8	2.25	1.125	0.1	0.1	0.05	0.01	0.01	0.008	Balance

### Sample Preparation

**FIGURE 1:**
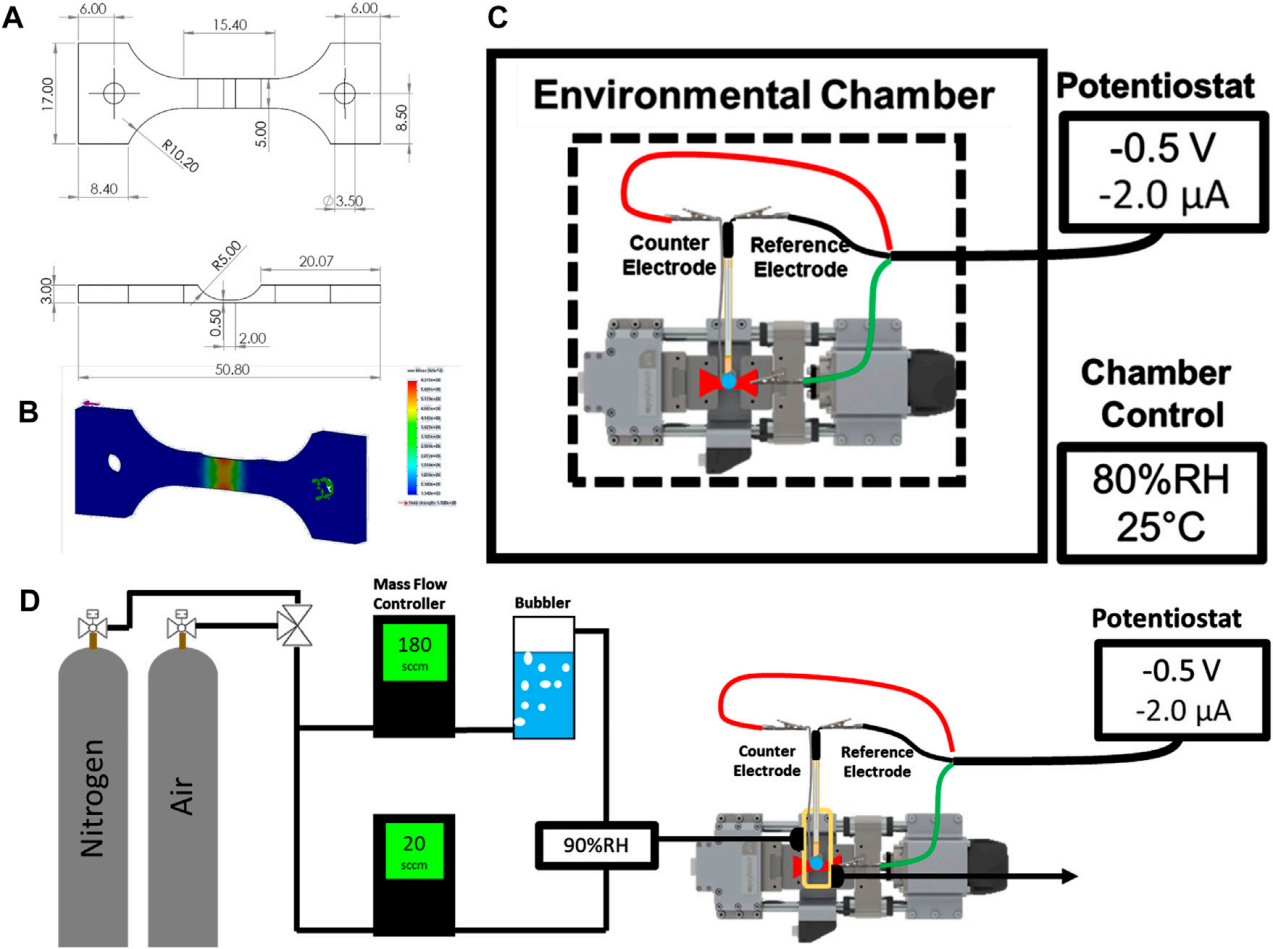
Schematic of the machined sample (mm) **(A)** and the corresponding static stress analysis **(B)**. Schematics for the environmental chamber configuration **(C)** and gas flow cell **(D)** are shown with labeled components.

UNS S13800 specimens were machined from bar stock into double-notch dogbone coupons. The dimensions and static stress analysis are shown in Figures 1A,B. A refining sequence of SiC paper (220P to 4000P) was used to grind the top surface down to a final notch thickness of 60–100 µm. Coupons were subsequently polished with 3 and 1 µm aqueous diamond slurries on a microfiber polishing pad. The polished coupons were washed with ultra-sonication in acetone, isopropyl alcohol, and 18 MΩ cm water for 5 min each. Cleaned coupons were dried under flowing nitrogen gas and masked to electrochemically constrain the active area above the notch region using polyester tape with a rubber-silicone adhesive blend.

### Characterization

Two different configurations were used to characterize the mechano-electrochemical behavior of UNS S13800. The first configuration is shown in Figure 1C and consisted of a compact load frame (Psylotech, Evanston, IL, United States) fixed to an aluminum breadboard inside an environmental chamber. The chamber was used to establish the temperature and relative humidity around the system. The relative humidity for a given temperature determines the partial pressure of water at that temperature, which determines the equilibrium water activity for hygroscopic salts above the efflorescence point (Shinohara et al., 2004; Van den Steen et al., 2016; Policastro et al., 2019). Relative humidity values of 95% and 80% (at 25°C) were used to maintain concentrations of approximately 0.6 and 4.6 M NaCl, respectively (Tang et al., 1986; Tang et al., 1997; Policastro et al., 2019). The coupon was mounted to the load frame with clamp grips containing garolite spacers to prevent ground loops between the load frame and the potentiostat controller. 0.6 or 4.6 M NaCl electrolyte (5–100 µl) was dispensed onto the active region of the specimen and a micromanipulator was used to position the reference and counter electrode in contact with the electrolyte. After positioning, the chamber was closed and allowed to stabilize for 30 min before initiation of the electrochemical experiments.

The second configuration (Figure 1D) was used to perform deoxygenated experiments with flowing gases without the use of a commercial environmental chamber. This setup, which employed a small plastic cell fixed to the specimen, enclosed the notch region of the specimen, but excluded the grip region. The cell was sealed to the specimen with marine epoxy (5200, 3M; St. Paul, MN, United States). Likewise, the reference and counter electrodes were fixed to the cell lid with marine epoxy. Threaded luer connectors were attached and sealed to the cell body for the gas inlet and outlet. The specimen was masked with polyester tape in the same way as the first setup. The relative humidity inside the small plastic cell was set by partitioning a fraction of the total gas flow through an aqueous bubbler to obtain both dry and saturated (100% RH) gas lines that were then recombined prior to entering the cell. The flow rate of each line was fixed with mass flow controllers to reach the specified relative humidity. Dry air and nitrogen were used as the flowing gas feed during oxygenated and deoxygenated experiments, respectively.

Both configurations used a Pt wire counter electrode and Ag/AgCl reference electrode. The reference electrode was checked against a saturated calomel electrode (SCE) prior to use and all potentials are reported against SCE (V_SCE_). Chronoamperometric experiments were conducted at −0.25, −0.5 and −0.8 V_SCE_. Mechanical load profiles conducted during chronoamperometric experiments were sequences performed with displacement control consisting of a tensile ramp, a static load, and a compressive ramp back to the pre-loaded state. Stress was measured with an inline load cell. A series of load sequences were utilized to examine strain rate effect and reproducibility.

Bulk polarization experiments were conducted in 0.6 and 4.6 M NaCl with a jacketed corrosion cell. A water circulator was used to maintain a steady temperature during experiments. Coupons were 1″ × 1″ × 1/8″ and masked to a 5/16″ diameter circle with polyester tape. A Pt mesh and SCE were used as counter and reference electrodes, respectively, with the reference electrode inside a fritted salt bridge containing saturated KCl to minimize ohmic drop. Potentiodynamic scans were performed cathodically (UNS S13800) from 0.02 V_OCP_ to −1.2 V_SCE_ and anodically (UNS A97075) −0.02 V_OCP_ to −0.6 V_SCE_ at a scan rate of 0.167 mV/s after an 18-h open circuit hold.

## Results

### Polarization

**FIGURE 2:**
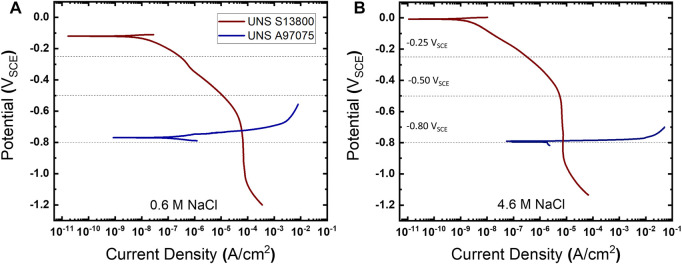
The polarization curves for UNS S13800 and UNS A97075 in **(A)** 0.6 and **(B)** 4.6 M NaCl at 25°C.

The cathodic polarization response for UNS S13800 is overlaid with the anodic response for UNS A97075 (AA7075T6) in Figure 2. These polarization curves were performed in cylindrical-bodied corrosion test cells (BioLogic; Knoxville, TN, United States) containing 300 ml of either 0.6 or 4.6 M NaCl. These NaCl concentrations are of interest in atmospheric corrosion as they correspond to sea water and the equilibrium NaCl droplet concentrations at 25°C/80% RH, respectively (Tang et al., 1997). UNS S13800 has a well-defined OCP between 0.0 and −0.1 V_SCE_ which is followed by the ORR activation region until approximately −0.5 V_SCE_, at which point the current transitions to diffusion limited ORR. In both cases onset of the hydrogen evolution reaction (HER) from water reduction occurs negative of the ORR limiting current, −1.0 V_SCE_ in 0.6 M NaCl and −0.9 V_SCE_ in the 4.6 M NaCl.

The potentials selected for the mechano-electrochemical experiments on the stainless-steel oxide were potentials at which ORR was under activation control (−0.25 V_SCE_), mixed control (−0.5 V_SCE_) and diffusion control (−0.8 V_SCE_). The latter is in close proximity to galvanic corrosion potentials observed from polarization experiments in Figure 2 (curve cross-over), which is the anticipated potential when UNS S13800 is coupled to UNS A97075. Anodic polarization of UNS A97075 displays rapid activation spanning over five orders of magnitude in corrosion rate in 50 and 30 mV for the dilute and concentrated NaCl solutions, respectively. The intersection of polarization curves has been used to predict both the galvanic corrosion potential and current. In Figures 2A,B the galvanic current is measured at 60 μA/cm^2^ and 7.4 μA/cm^2^ in 0.6 and 4.6 M NaCl, respectively.

The polarization curves in Figure 2 suggest UNS S13800-UNS A97075 galvanic couples in a 1:1 cathode to anode area ratio would be under cathodic control. This is due to the ORR diffusion limited behavior near the galvanic potential, which results in relative potential insensitivity of the ORR rate in contrast to the much smaller Tafel slope of UNS A97075. Additionally, the predicted galvanic corrosion rate from polarization curves in Figure 1 is ∼8.1 times larger in 0.6 M NaCl (60 μA/cm^2^) than 4.6 M NaCl (7.4 μA/cm^2^). This is in accord with the limiting diffusion current (*i*_*l*_) equation:

$$i_{l}=\frac{\mathrm{nF}D_{O_{2}}C_{O_{2}}}{\delta }$$ (1)

Equation 1 indicates the limiting current density is proportional to the Faraday constant (*F*), the reaction equivalent (*n*), oxygen diffusivity ( $D_{O_{2}}$ ) and oxygen concentration ( $C_{O_{2}}$ ) and inversely proportional to the diffusion thickness layer (*δ*). The oxygen saturation concentrations for the two NaCl concentrations reside at 214 and 68 µmol O_2_/kg H_2_O for 0.6 and 4.6 M NaCl, respectively(Millero et al., 2002). The oxygen diffusivities have been reported to be 1.97 × 10^−5^ and 1.40 × 10^−5^ cm^2^/s for 0.6 and 4.6 M NaCl at 25°C, respectively(Mizuno and Kelly, 2013). These differences give a $D_{O_{2}}C_{O_{2}}$ ratio of 118:10, an 11 fold difference in driving force for oxygen diffusion, which is similar to the limiting current density ratio.

### Chronoamperometry

**FIGURE 3:**
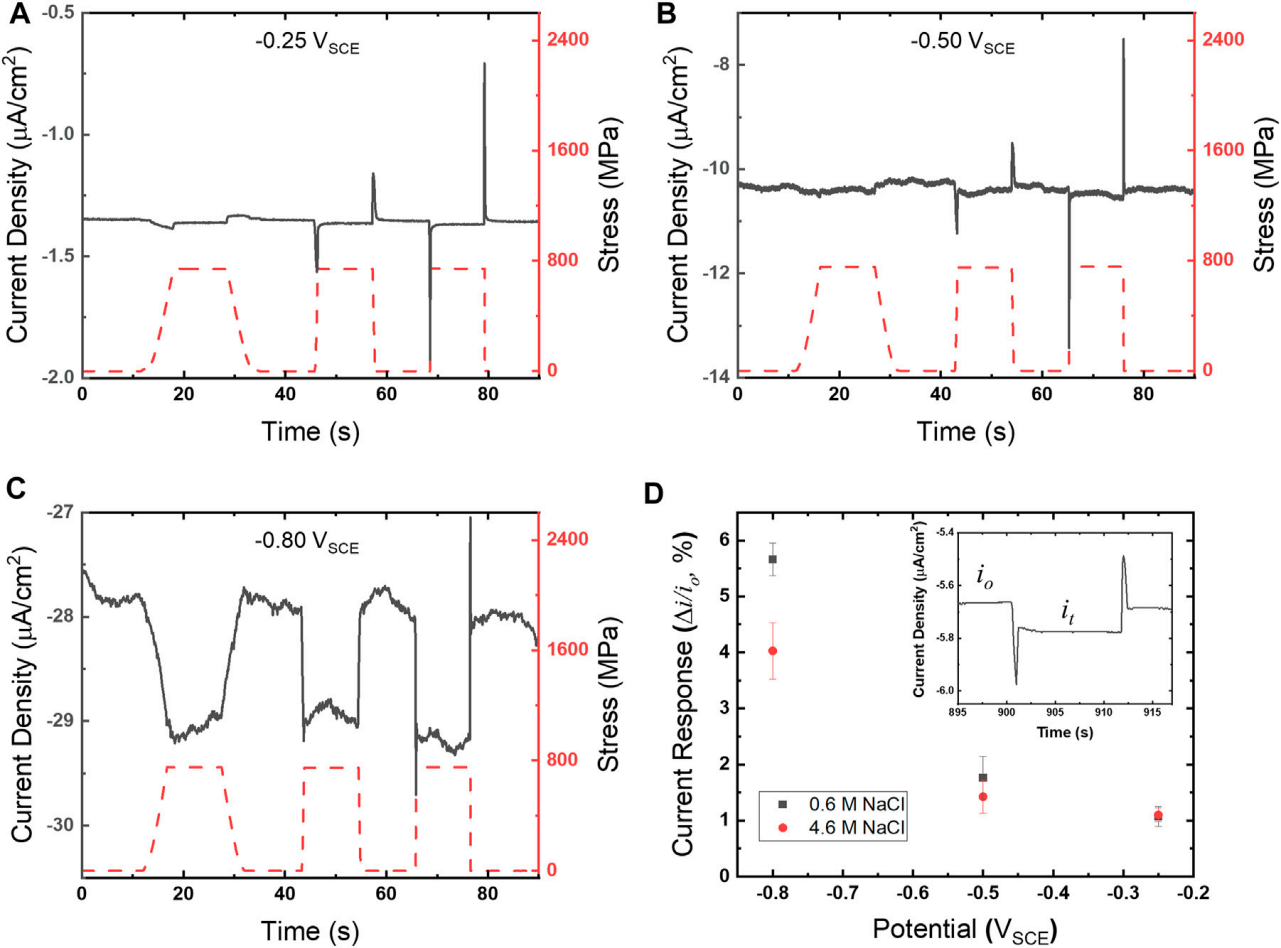
Chronoamperograms (gray solid line, left ordinate) and corresponding stress profiles (red dashed line, right ordinate) for UNS S13800 with 25 μl of 4.6 M NaCl at 25°C and 80% RH for **(A)** −0.25 V_SCE_, **(B)** −0.50 V_SCE_ and **(C)** −0.80 V_SCE_. Strain rates for **(A–C)** follow a sequence of 0.0002, 0.002 and 0.02/s. The current responses for 4.6 and 0.6 M NaCl determined from Eq. 1 are shown in **(D)** as a function of potential.

Chronoamperograms for UNS S13800 were collected utilizing the experimental configuration shown in Figure 1C. Representative chronoamperograms, along with the overlaid concurrent stress profile (right ordinate), are shown in Figures 3A–C. The stress reaches values of 700–800 MPa, residing well within the elastic regime for UNS S13800 which is reported to be ∼1,450 MPa (Tyler et al., 1991). Tensile holds were examined at varied strain rates that included some combination of 0.00002, 0.0002, 0.002 and 0.02/s. The measured current transients showed an increased cathodic current during the strain ramp. The cathodic current stabilized during the tensile hold and then returned to baseline values upon release to the pre-loaded state. Additionally, cathodic and anodic current peaks demarcated initiation and termination of the response for the faster strain rates. Quantification of the current response to tension was determined by the difference in current under tension (*i*_*t*_) and the baseline current (*i*_*o*_) using the following equation:

$$R=\frac{i_{t}-i_{o}}{i_{o}}\times 100$$ (2)

The current response for UNS S13800 under 0.6 and 4.6 M NaCl is shown in Figure 3B with an inset displaying *i*_*t*_ and *i*_*o*_ regions of the current response. Values for *i*_*t*_ and *i*_*o*_ were determined from the average of current values over the 5 s just prior to tensile and compressive ramping, respectively. The current response increased monotonically with more negative potentials. Measurements performed in dilute NaCl solutions displayed a larger current response when the ORR was under diffusion limited control.

**FIGURE 4:**
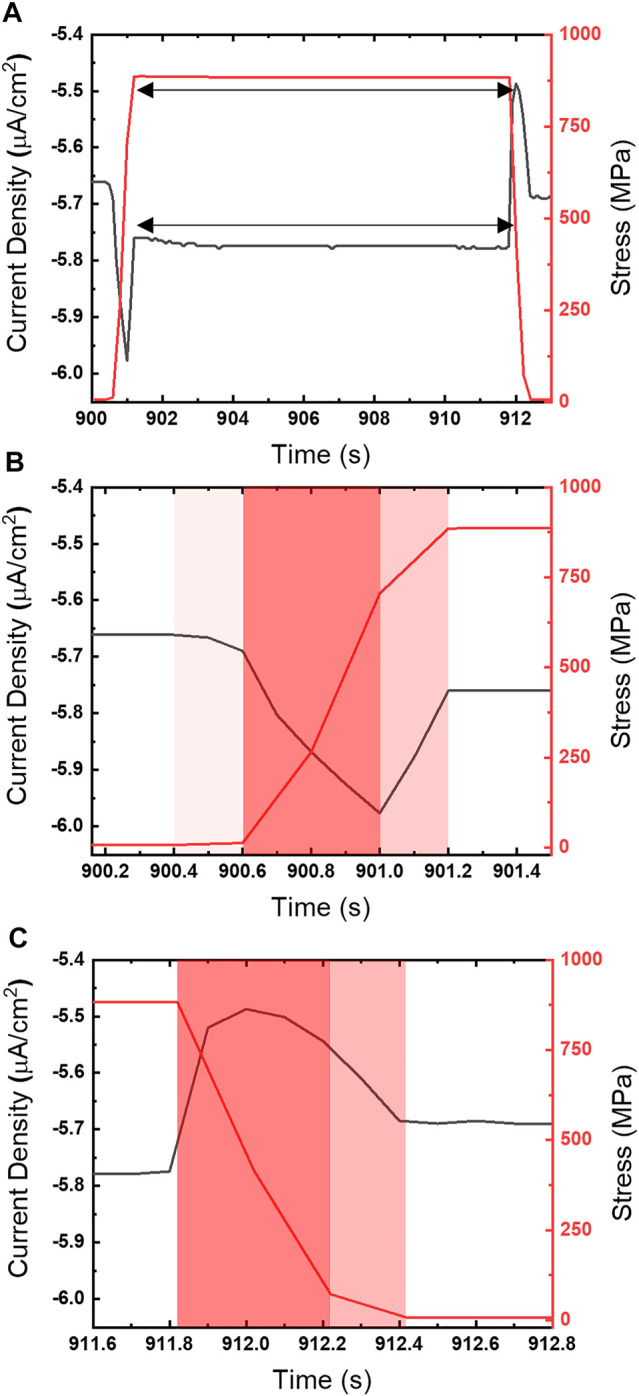
**(A)** Chronoamperogram (left ordinate) overlaid with stress profile (right ordinate) for UNS S13800 under 25 μl of 4.6 M NaCl at 25°C and 80% RH at −0.50 V_SCE_. The current response and stress profile to **(B)** tensile and **(C)** compressive ramping are also shown on a finer time scale. The nominal strain rate during ramping is 0.002/s.

Close examination of the chronoamperometry in comparison with the stress profiles (Figure 4) reveal the current peaks observed during high strain rates closely follow tension ramping and relaxation steps. The cathodic peak (Figure 4B) can be split into a cathodic (ascending) and anodic (descending) region that correspond with the transition from the strain rate maximum (red) to a declining strain rate (pink) just prior to the static hold. The response during relaxation (Figure 4C) does not exhibit a distinct correlation between current direction and strain rate changes. In contrast, the anodic peak that occurs once the strain rate changes to relaxation, rapidly rises over the course of 0.2 s to a maximum value, but then turns cathodic while the strain ramp is still at 0.002/s.

## Discussion

Identifying the reaction processes affected by mechanical load profiles is important for interpretation of mechano-electrochemical responses on oxide covered structural alloys. From Figure 3 the two primary amperometric responses include a shift in the reduction current during tensile holds and current peaks during strain ramps with electrochemical polarity (cathodic and anodic) that corresponds to strain direction (tensile and compressive). Although ORR is recognized as the primary reductive reaction in oxygenated environments for potentials this work examined, additional contributions could arise from oxide dynamics or even HER. Importantly, HER is also occurring at the most negative potential examined, albeit at rates 1–2 orders of magnitude smaller than ORR based on extrapolation of the HER Tafel slope in Figure 2.

**FIGURE 5:**
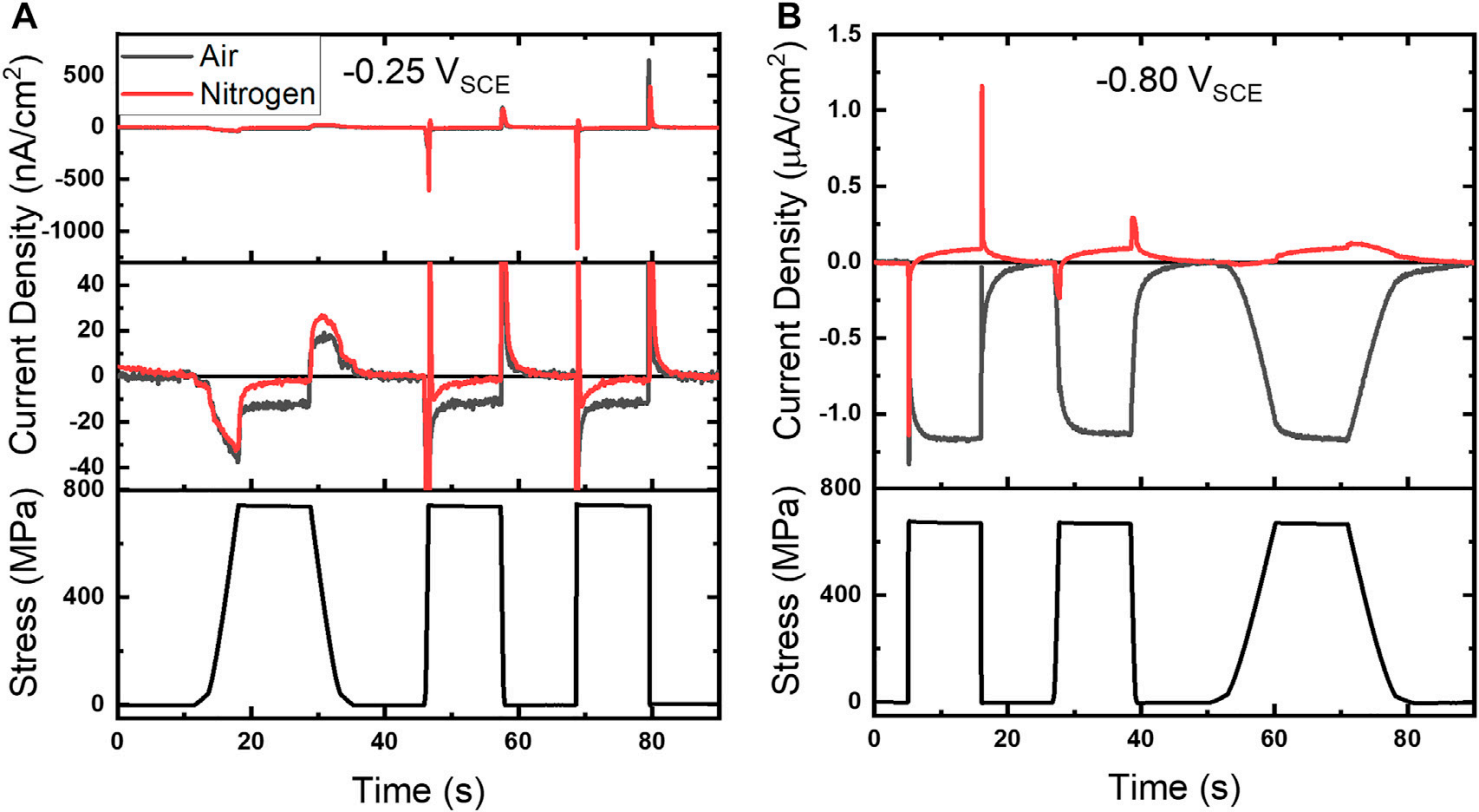
**(A)** Baseline subtracted chronoamperograms under 25 µl of 4.6 M NaCl in air and nitrogen atmospheres along with corresponding stress profile at **(A)** −0.25 V_SCE_ and **(B)** −0.80 V_SCE_. A finer scale chronoamperogram is shown in the middle plot of **(A)**.

A modified experimental configuration shown in Figure 1D was implemented to switch between oxygenated (air) and deoxygenated (N_2_) atmospheres to better identify ORR contributions. This strategy allowed for direct comparison of amperometric baseline shifts and current peaks in oxygenated and deoxygenated environments, shown in Figure 5 for −0.25 and −0.80 V_SCE_, along with concurrent stress profiles (Figure 5 bottom plots). A fine scale view of the chronoamperograms at −0.25 V_SCE_ is shown in the middle plot of Figure 5A. Comparison in these environments reveals the current response in N_2_ (red line) is close to the baseline during the tensile hold, while a clear shift was observed in air (gray line). The N_2_ ramp regions at −0.25 V_SCE_ display current peaks that are aligned and of the same approximate magnitude as the corresponding oxygenated experiments. However, an anodic oscillation is observed following the cathodic peak at both 0.002 and 0.02/s. At the more negative potential, -0.80 V_SCE_, the deoxygenated environment not only suppresses ORR but an anodic current, with respect to the baseline current, is observed for each strain rate. These results, as a whole, demonstrate the current shift during tensile holds (*i*_*t*_) is primarily attributable to ORR while the current peaks are likely a conflated response to ORR and oxide dynamics.

**FIGURE 6:**
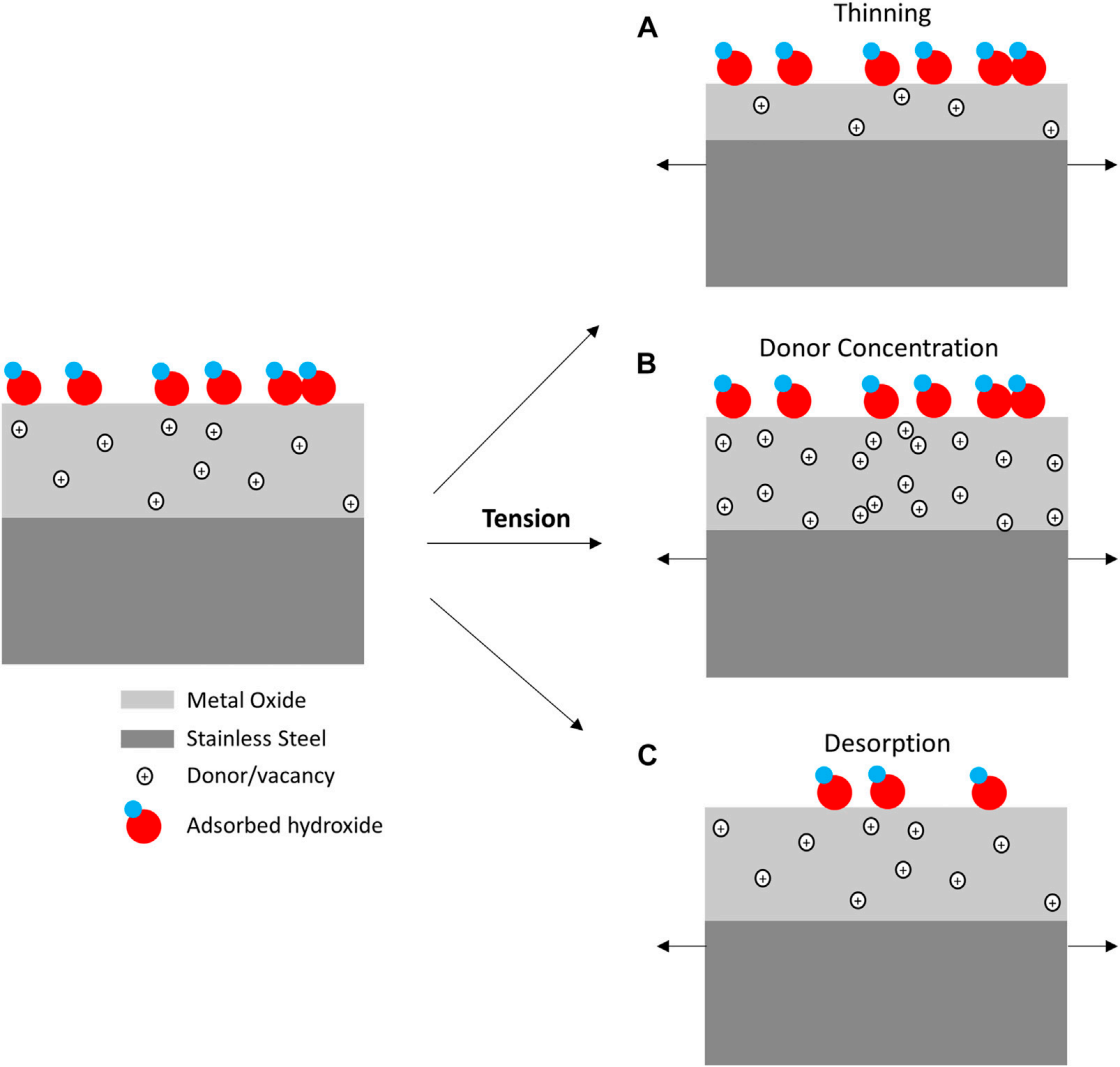
Schematic of mechanisms that may impact reductive reaction rates going from an unstressed to tensile state include conductance changes from **(A)** oxide thinning and **(B)** donor concentration/mobility changes as well as rate changes from **(C)** ad/desorption phenomena.

Surface oxides play an important role in dictating charge transfer on corrosion resistant materials. Therefore, oxide dynamics under mechanical loads are important in understanding the amperometric behavior reported in this effort (Moffat et al., 1992). Specific oxide behavior is often quite difficult to definitively assign as this umbrella term may include changes in charge carrier concentration/mobility, oxide thickness, and electroactive adsorbed species, as shown in Figure 6. Even though these mechanisms are discussed and depicted separately in Figure 6, it is important to recognize they are not necessarily mutually exclusive.

The increased ORR current during tensile holds is consistent with a drop in oxide resistance. Moreover, this resistance change appears fully reversible with a return to the current baseline values after decay of the current peak. Although the reductive potentials examined in this study for galvanic relevance produce oxides with distinct behavior with respect to those typically observed in repassivation studies (Sidane et al., 2011; Nazarov et al., 2019), some efforts have looked at charge carrier density across anodic and cathodic potentials (Moffat et al., 1992; Vignal et al., 2001). Vignal et al. have considered the impact of mechanical stress on the conductivity of the oxide in the context of a bilayer oxide structure and aging. Their work showed that while acceptor density, i.e., inner p-type chromium oxide conductivity, of aged oxides is not impacted by mechanical stress, passive films formed in the presence of elastic stress exhibited a discernible increase in donor concentration with increase tensile stress. The amperometric results reported here correlate with the latter in both preparation and tension response. That is, our experiment examined polished samples without a passive film growth procedure, and their behavior correlates with higher conductance under tensile stress, manifesting as an increased ORR current.

Recent density functional theory calculation have also indicated that tensile strain on chromium oxide introduces both Cr and O vacancies (Mi et al., 2018). These vacancies were predicted to introduce energy states below the conduction band, with Cr vacancies causing a significant decrease in the band gap. These model predictions are consistent with our results that showed an increased charge transfer for ORR during applied tensile strain. Similar modeling work has also underscored the importance of vacancy formation during applied tension to reconcile theoretical and experimental fracture behavior of chromia (Li et al., 1999; Hirota et al., 2002; Pang et al., 2007; Islam et al., 2017). That being said, structural alloy surface oxides have been shown to span epitaxial crystallinity to amorphous regions as chromium content increases above 11% (Olsson and Landolt, 2003). Surface oxides have also been recently shown to demonstrate rapid surface diffusion with liquid-like behavior that brings into question passive film plasticity (Yang et al., 2018).

Additionally, a thinned oxide layer, proposed by the film rupture-repassivation model, is congruent with greater conductance during tensile stress (Song et al., 2006). In this model, thinning of the oxide is thought to occur while the oxide is undergoing elastic deformation, consistent with mechanisms of defect formation in response to strain, uptake of chloride ions and subsequent displacement of hydroxide that releases Fe^3+^ ions, prior to crack nucleation and growth (Marcus et al., 2008). Importantly, several efforts have shown chloride does not impact the inner passivation layers; only the iron rich outer layers produced in chloride environments (Natishan et al., 2012; Natishan, 2018; Wang et al., 2020). This view is consistent with other groups that have argued chloride adsorption occurs at defect sites, which lowers the energy barrier for oxidation producing lateral heterogeneity that manifest as morphological instabilities (Sato, 1971; Zhang et al., 2018; Ramanathan and Voorhees, 2019). While cracking has been associated with anodic repassivation current from electrolyte exposure of new metal surface area, more facile charge transfer should result from a thinner resistive inner layer. This reasoning implies a thinning mechanism would only apply to the current shift during tensile holds and excludes current peak behavior during tensile ramps.

One contention with either perspective of the resistive oxide response to tension (Figure 6A) would be the larger current response to tensile stress in the diffusion limited region (−0.8 V_SCE_). ORR in this potential window is considered to proceed at rates faster than transport occurs, which should not be influenced by sample stress. However, previous efforts have demonstrated hysteresis in cyclic polarization experiments with discernible current difference between −0.6 and −0.8 V_SCE_ that were attributed to oxide reduction during the tail end of the cathodic sweep (Alexander et al., 2018).

The amperometric response during strain ramping is unique in that a reductive current peak occurs during tensile ramping with the converse being true during relaxation, despite many oxide growth, dissolution and defect/donor reactions being oxidative in nature (Seyeux et al., 2013). The current polarity and time scale is not consistent with repassivation, which should produce anodic current peaks on the order of 1–10 ms upon applied tension (Kolman and Scully, 1999). While some of this current may be a consequence of increased ORR, these peaks are clearly observed in the absence of oxygen. Integration of the peaks from the deoxygenated chronoamperogram in Figure 5A produced a charge between 5 and 7 nC for each peak. This similarity in peak charge (Coulombs) across ramp rates appears to be a capacitance shift. This is not surprising considering the potential dependent capacitance values of passive films reported previously (Jovancicevic and Bockris, 1986; Santamaria et al., 2015). This capacitance includes changes in the oxide, such as ion release, phase changes and adsorption, all of which will impact the double layer, with the former displaying potential dependence from formal passive film formation potentials to those examined in this study. Redox reactions within the passive film include reduction of Fe^3+^ to Fe^2+^ during cathodic peaks, with the reverse reaction occurring during anodic peaks. Similarly an increase in specific desorption of anionic species (e.g., OH^−^) from an oxide during tensile stress will give rise to a cathodic peak, with adsorption producing an anodic peak (McCafferty, 2010). The time scale for these reactions has been shown to be on the order of a minute for pH stepping experiments with iron (Jovancicevic and Bockris, 1986).

ORR on stainless steels and iron is generally recognized as proceeding with adsorption of molecular oxygen onto the surface followed by reduction steps and product dissociation (Jovancicevic and Bockris, 1986; Calvo and Schiffrin, 1988; Ng et al., 2020). ORR on oxide covered stainless steels in chloride electrolytes has been shown to follow the four electron pathway as well as a mix of two and four electron pathways (Le Bozec et al., 2001; Alexander et al., 2018). Pre-reduced stainless steels were shown to follow the four electron pathway, while polished and passivated surfaces include a mix of 2 and 4 electron pathways. Recent work has also shown that transport limitations can impact reaction pathway, shifting from the four-electron pathway to mixed response as the diffusion layer decreases (Alexander et al., 2018). These reaction pathways imply a shift in adsorbed species as well as their reactivity. The amperometric response to tension in Figure 3 may therefore reside in reaction pathway valence shifts or adsorbate shifts that increase site availability, both of which may increase ORR current.

In the context of chloride concentration effects (Figure 3D), chloride has been previously shown to decrease iron content in stainless steel passive films (Kocijan et al., 2007). Experimental data and theoretical calculations have examined ORR at both Cr_2_O_3_ and Fe_2_O_3_ rich surfaces and found ORR proceeds preferentially at Fe_2_O_3_ (Le Bozec et al., 2001; Ng et al., 2020). Hematite essentially reduces the overpotential required for ORR, with respect to chromia. Density functional theory calculations predict hydroxide-terminated hematite surfaces to exhibit the lowest ORR overpotential (Ng et al., 2020). Results for several pretreated stainless steel surfaces (i.e., polished, pre-reduced, chemically treated and electrochemically passivated) have also found that Fe^2+^ enrichment, by evaluation of Fe^2+^:Fe^3+^ content in the oxide, reduces the ORR overpotential (Le Bozec et al., 2001). While chloride adsorption and incorporation have not been shown to result in film thickness differences for typical marine corrosion chloride concentrations (<1 M NaCl), atmospheric studies have demonstrated thinning of the iron oxide outer layer under concentrated NaCl near the deliquescence point (Jung et al., 2012).

This suggests a possible explanation for the chloride-dependent differences plotted in Figure 3D, in which chloride tempers the amperometric response to tensile stress most significantly at −0.8 V_SCE_. At this potential, reduction of Fe^3+^ to Fe^2+^ is expected to be more significant and hence the effect of chloride-induced loss of reducible iron may be more pronounced. The polarization resistance values of the stabilized surface oxides in bulk solutions of 0.6 and 4.6 M NaCl were observed to be 1.6 and 5.2 MΩ, respectively. These values were determined from the linear region, OCP ±5 mV, of the polarization data plotted in Figure 2. The increase in polarization resistance with chloride concentration may arise from selective displacement of hydroxide groups and the dissolution of Fe^3+^ described above (Marcus et al., 2008). This conductance and overpotential relationship with chloride may be responsible for [Cl^−^] dependent behavior observed herein.

## Summary

In summary, this work has examined the role of tensile stress on oxygen reduction rates for UNS S13800 in chloride electrolytes. Deoxygenated amperometry was utilized to discern ORR contributions to current shifts during static tensile holds and dynamic current during strain ramping. The current response of the UNS S13800 to tensile stress is shown to increase at more negative potentials, with the strongest response occurring at −0.8 V_SCE_, a relevant potential for galvanic corrosion with aluminum alloys. This behavior was attributed to decreased oxide resistance from some combination of changes in donor concentration, oxide thinning, cracking and/or adsorption effects. The oxygen free current peaks during strain ramps were attributed to changes in specific adsorption and redox reactions with metal cations. These results highlight the importance of mechanical stress effects on corrosion reaction rates. Further distinction of the mechanism at play in this system will require examination of the surface oxide structure by spectroscopic or alternating current techniques.

## Data Availability

The raw data supporting the conclusion of this article will be made available by the authors, without undue reservation.
